# Editorial of Special Issue “Metabolomic Analysis in Health and Diseases”

**DOI:** 10.3390/jcm10163491

**Published:** 2021-08-07

**Authors:** Masahiro Sugimoto

**Affiliations:** 1 Research and Development Center for Minimally Invasive Therapies, Tokyo Medical University, Shinjuku, Tokyo 160-8402, Japan; mshrsgmt@tokyo-med.ac.jp or mshrsgmt@gmail.com; Tel.: +81-235-29-0528; Fax: +81-235-29-0574; 2 Institute for Advanced Biosciences, Keio University, 246-2 Mizukami, Kakuganji, Tsuruoka, Yamagata 997-0052, Japan

Metabolomics, a quantitative omics technology that simultaneously profiles hundreds of metabolites, has been used to explore new biomarkers and elucidate the metabolic pathways perturbed by various stimuli at a system level. The tool enables us to evaluate systemic health status and metabolic diseases using high-dimensional metabolomics data. In this Special Issue, we published current metabolomics topics related to health and diseases. Nine papers were published in total.

In five studies, clinical biofluid samples to explore biomarkers for differentiation of diseases and monitoring of treatment response were analyzed. Shimizu et al. have attempted to discover new biomarkers for ophthalmic diseases. They used liquid chromatography−time-of-flight mass spectrometry (LC-TOFMS) to quantify charged metabolites in serum. They analyzed serum samples collected from patients with three major forms of uveitis, including Behҫet’s disease, sarcoidosis, and Vogt–Koyanagi–Harada disease. Results revealed that the combination of 24 metabolites showed a potential to differentiate one of the diseases from other diseases [1]. Ishikawa et al. analyzed the relationship between the maximum standard update values (SUV_max_) of ^18^F-fluorodeoxyglucose positron emission tomography (PET)/computed tomography (CT) and salivary metabolites quantified using capillary electrophoresis-TOFMS (CE-TOFMS). The identified metabolites showed a potential to detect patients with high SUV_max_ values [2]. Kawanishi et al. evaluated the effect of prosthetic treatment on patients with *Candida albicans* and found an improvement in saliva flow rate [3]. Yasuoka et al. conducted CE-TOFMS-based metabolomics of saliva samples collected from patients with head and neck cancers who underwent chemotherapy [4]. Kuroiwa et al. analyzed amino acids in human plasma samples and found that branched-chain amino acid concentrations correlated with brown adipose tissue density [5].

An *in vivo* study and two *in vitro* studies were published. To et al. evaluated the relationship between cognitive function and salivary metabolomic profiles analyzed using CE-TOFMS. Cognitive impairment was induced by decreased expression of hippocampal brain-derived neurotrophic factor (BDNF) [6]. Sakagami et al. investigated the anti-inflammatory action of the alkaline extract of Sasa sp. (SE) leaves. The extract has been used for the treatment of oral disease, and multi-omics analyses using transcriptomics and metabolomics revealed the changes in the pathway level of the extract on human gingival fibroblast cells [7]. Kato et al. conducted a CE-TOFMS-based metabolomics analysis to capture a holistic view of the metabolic pathways of oral keratinocytes and fibroblasts. They compared the metabolomic profiles under various hypoxic culture conditions in the cells [8].

A study proposed a computational method to enhance the quality of metabolomic analysis of clinical samples. Saito et al. developed a quality assessment pipeline for the CE-TOFMS data. Although most of such analyses had focused only on targeted data, they devised analyses for untargeted analytical data. They evaluated their methods using large-scale datasets of urine and plasma samples collected in a cohort study that included 3000 participants [9].

The studies in this Special Issue included both basic research and the development of a new method for metabolomics applications. More rigorous validations are necessary for biomarker studies before they are used under clinical settings, and the understanding of underlying molecular mechanisms based on in vitro and in vivo studies would help identify biomarkers. Finally, the quality assessment would compensate for the shortcomings arising from the analytical fragility of metabolomics.

As the Guest Editor, I thank the reviewers for their professional comments and the JCM Editorial Office for their robust support. I believe the readers of this Special Issue will find the articles very useful.
